# Awareness of Prediabetes — United States, 2005–2010

**Published:** 2013-03-22

**Authors:** YanFeng Li, Linda S. Geiss, Nilka R. Burrows, Deborah B. Rolka, Ann Albright

**Affiliations:** Div of Diabetes Translation, National Center for Chronic Disease Prevention and Health Promotion, CDC

In 2010, approximately one in three U.S. adults aged ≥20 years (an estimated 79 million persons) had prediabetes, a condition in which blood glucose or hemoglobin A1c (A1c) levels are higher than normal but not high enough to be classified as diabetes (1). Persons with prediabetes are at high risk for developing type 2 diabetes, which accounts for 90%–95% of all cases of diabetes. Each year, 11% of persons with prediabetes who do not lose weight and do not engage in moderate physical activity will progress to type 2 diabetes during the average 3 years of follow-up (2). Evidence-based lifestyle programs that encourage dietary changes, moderate-intensity physical activity, and modest weight loss can delay or prevent type 2 diabetes in persons with prediabetes (2). Identifying persons with prediabetes and informing them about their increased risk for type 2 diabetes are first steps in encouraging persons with prediabetes to make healthy lifestyle changes. However, during 2005–2006, only approximately 7% of persons with prediabetes were aware that they had prediabetes (3). To examine recent changes in awareness of prediabetes and factors associated with awareness among adults aged ≥20 years, CDC analyzed data from the National Health and Nutrition Examination Survey (NHANES). This report describes the results of that analysis, which indicated that, during 2009–2010, approximately 11% of those with prediabetes were aware of their condition. Furthermore, during 2005–2010, estimated awareness of prediabetes was <14% across all population subgroups, different levels of health-care access or use, and other factors. In the United States, persons with prediabetes, including those with regular access to health care, might benefit from efforts aimed at making them aware that they are at risk for developing type 2 diabetes and that they can reduce that risk by making modest lifestyle changes. Efforts are needed to increase awareness.

NHANES is an ongoing, stratified, multistage probability sample of the noninstitutionalized U.S. civilian population. It includes personal interviews, medical examinations, and laboratory measurements (4). This analysis was conducted using data from three sampling cycles of NHANES, with examination response rates of approximately 77% for 2005–2006, 75% for 2007–2008, and 77% for 2009–2010 (4). Of 6,938 nonpregnant participants aged ≥20 years assigned to a morning fasting session, 6,771 had valid values for both fasting plasma glucose (FPG) and A1c tests. After excluding those with self-reported diabetes (n = 834) and those with undiagnosed diabetes (FPG ≥126 mg/dL or A1c ≥6.5%) (n = 310), a total of 2,603 participants with prediabetes (FPG 100–125 mg/dL or A1c 5.7%–6.4%) were identified. Adult participants were classified as being aware of their prediabetes if they 1) answered “yes” to the question, “Have you ever been told by a doctor that you have prediabetes, borderline diabetes, impaired fasting glucose, impaired glucose tolerance, or that your blood sugar is higher than normal but not high enough to be called diabetes or sugar diabetes?” or 2) reported having prediabetes when asked whether they had diabetes. The prevalence of prediabetes awareness was compared across selected sociodemographic characteristics, health-care access or use characteristics, and other factors. Sociodemographic characteristics included age group, race/ethnicity, sex, education level, and poverty-to-income ratio (PIR).* Health-care access or use characteristics included having any health insurance or other health-care coverage at time of interview, number of doctor visits in the past year, and having a usual source of care (defined as those reporting having a place they usually go to for care that was a doctor’s office or clinic as opposed to no place or a hospital outpatient or emergency department). Other characteristics examined included family history of diabetes, reported current use of medication for hypertension or hypercholesterolemia, and body mass index (BMI) obtained from measured height and weight and classified as normal weight (BMI <25.0 kg/m^2^), overweight (BMI 25.0–29.9 kg/m^2^), and obese (BMI ≥30.0 kg/m^2^). Analyses were performed with sampling weights, which account for the complex sampling design. Age-adjusted estimates were calculated by the direct method using the 2000 U.S. standard population. T-tests were used to examine the differences between subgroups.

During 2005–2010, the percentage of persons aged ≥20 years with prediabetes who were aware of their prediabetes remained low but was slightly higher during 2009–2010 (11.1%) than during 2005–2006 (7.7%, p=0.04) (Table). During 2005–2010, the prevalence of prediabetes awareness was lower among persons aged 20–44 years (5.1%) compared with persons aged 45–64 years (10.0%) and those aged ≥65 years (11.9%; both p<0.002) (Table). Age-adjusted prevalence of prediabetes awareness was lower among persons with less than a high school education (4.9%) compared with those with greater than a high school education (8.7%, p=0.003). It was higher among those overweight (7.9%) and those obese (9.9%) compared with among those of normal weight (4.3%, p=0.045 and p=0.004 respectively). Also, it was higher among those with a family history of diabetes compared with those without (10.4% versus 6.2%, p=0.001), among those reporting taking either hypertension or hypercholesterolemia medication compared with those not taking such medication (13.9% versus 6.1%, p=0.01), among those with health insurance or other coverage at time of interview compared with those without (8.4% versus 4.7%, p=0.008), and in those reporting a usual source of care that was either a clinic or doctor’s office (8.9%) compared with those without a usual source of care or those who received care in a hospital outpatient or emergency department (4.4%, p=0.01). Compared with those having fewer than two doctor visits in the past 12 months (5.4%), persons visiting doctors more than once were more likely to be aware of their prediabetes (9.0% for those having two or three visits, p=0.048, and 10.5% for those having four or more visits, p=0.008). No statistically significant association was observed between prediabetes awareness and sex, race/ethnicity, or PIR group.

What is already known on this topic?

Although an estimated one third of U.S. adults aged ≥20 years have prediabetes, during 2005–2006, only about 7% of them were aware that they had prediabetes. Evidence-based lifestyle-change programs that encourage dietary changes, moderate-intensity physical activity, problem-solving skills, and modest weight loss can delay or prevent type 2 diabetes among those with prediabetes. Interventions to promote identification and improved awareness of prediabetes are key first steps to implementing such programs for persons at high risk for type 2 diabetes.

What is added by this report?

The proportion of U.S. adults with prediabetes aged ≥20 years who were aware that they had prediabetes remained low, with only 11% reporting during 2009–2010 that they had prediabetes. Further, awareness of prediabetes was low (<14%) regardless of educational level, income level, coverage by health insurance or other kind of health-care plan, or health-care use.

What are the implications for public health practice?

Persons with prediabetes, including persons with regular access to health care, might benefit from efforts aimed at making them aware that they are at risk for developing type 2 diabetes and that they can reduce their risk by making modest lifestyle changes.

## Editorial Note

This report indicates that the proportion of U.S. adults with prediabetes who report being told they have prediabetes remained low, with only 11.1% reporting during 2009–2010 that they have prediabetes. It also indicates awareness of pre-diabetes was low (<14%) across all population subgroups and different levels of health-care access or use and other factors. Thus, interventions to promote identification and increased awareness of those with prediabetes are needed to encourage adoption of type 2 diabetes prevention strategies, particularly among groups known to be at high risk for type 2 diabetes.

Risk factors for prediabetes and type 2 diabetes include being aged ≥45 years; being overweight or obese; having a family history of diabetes; being of African American, Hispanic/Latino, American Indian, Asian American, or Pacific Islander race/ethnicity; having given birth to a baby weighing ≥9 pounds (4,082 g) or having a history of gestational diabetes; and being physically active <3 times a week (5–7). The American Diabetes Association has recommended that testing for prediabetes and diabetes be considered for adults with risk factors (7). Persons unaware of their risk should discuss their risk with their health-care provider and can take an online quiz to assess their risk for prediabetes.†

Evidence-based lifestyle programs aimed at increasing physical activity, improving diet, and achieving moderate weight loss (i.e., approximately 7% of total body weight) among those with prediabetes and BMI ≥24.0 kg/m^2^ can prevent or delay type 2 diabetes (2). The CDC-led National Diabetes Prevention Program,§ a public-private partnership of community organizations, private insurers, employers, health-care organizations, and government agencies, supports the nationwide implementation of evidence-based, lifestyle-change programs in the community that promote modest weight loss, good nutritional practices, increased physical activity, and problem-solving skills among persons at high risk for developing type 2 diabetes. Also, the National Diabetes Education Program,¶ a partnership of the National Institutes of Health and CDC, provides resources to reduce the risk for type 2 diabetes, including resources such as “Small Steps. Big Rewards. Your Game Plan to Prevent Type 2 Diabetes” and “Just One Step,” which provide helpful tips in making lifestyle changes.

The findings in this report are subject to at least five limitations. First, NHANES participants with impaired glucose tolerance (based on 2-hour oral glucose tolerance test values of 140–199 mg/dL) were not included in the definition of prediabetes; had they been included, the overall estimate of awareness during 2009–2010 would have been 10.0% rather than 11.1%. Second, data on prediabetes awareness and most other characteristics were self-reported and might be subject to recall bias. Third, because NHANES surveys only the noninstitutionalized U.S. civilian population, military personnel and persons residing in nursing homes and other institutions are not included. Fourth, the NHANES examination response rates were approximately 75%; the actual level of awareness might be higher or lower if nonparticipants differed systematically from participants. Finally, results of the laboratory tests that were used to define prediabetes vary within persons across time, blood specimen, and laboratory analysis. However, on average, the single pair of test results obtained for a participant in this study would be expected to approximate the mean values for similar persons in the U.S. population. Compared with FPG, A1c has less within-person variability (8).

Although diabetes prevalence is increasing in the United States (9), type 2 diabetes can be prevented or delayed among those who are at high risk by modest weight loss, good nutritional practices, and increased physical activity. Because the vast majority of persons with prediabetes are unaware of their condition, identification and improved awareness of prediabetes are critical first steps to encourage those with prediabetes to make healthy lifestyle changes or to enroll in evidence-based, lifestyle-change programs aimed at preventing type 2 diabetes.

## Figures and Tables

**TABLE:** Crude and age-adjusted prevalence of prediabetes awareness* among adults aged ≥20 years with prediabetes† — National Health and Nutrition Examination Survey, United States, 2005–2010

Characteristic	No. with prediabetes§	Crude¶		Age-adjusted**	
	
		%	(95% CI)	%	(95% CI)
**Total (2005–2010)**	**2,603**	**8.9**	**(7.7–10.2)**	**7.8**	**(6.6–9.3)**
2005–2006	626	7.7	(5.8–10.1)	6.5	(4.7–9.0)
2007–2008	957	7.7	(6.0–9.9)	6.6	(4.9–8.8)
2009–2010	1,020	11.1	(9.0–13.6)	10.1	(7.8–13.0)
**Age group (yrs)**					
20–44	759	5.1	(3.3–7.9)	NA	—
45–64	1,040	10.0	(8.3–12.0)	NA	—
≥65	804	11.9	(9.3–15.0)	NA	—
**Race/Ethnicity**					
White, non-Hispanic	1,293	9.1	(7.6–10.8)	7.6	(6.0–9.4)
Black, non-Hispanic	489	8.3	(5.8–11.7)	7.9	(5.4–11.4)
Mexican American	475	5.6	(3.8–8.0)	7.0	(5.0–9.7)
Other††	346	11.2	(7.1–17.1)	11.0	(7.2–16.3)
**Sex**					
Men	1,423	7.6	(6.0–9.4)	6.6	(5.2–8.3)
Women	1,180	10.6	(8.4–13.2)	9.9	(7.3–13.2)
**Education**					
<High school	800	6.0	(4.4–8.2)	4.9	(3.6–6.7)
High school	653	8.6	(6.2–11.8)	8.3	(5.6–12.2)
>High school	1,144	10.3	(8.6–12.1)	8.7	(7.2–10.6)
**Poverty-to-income ratio**					
Poverty (<100%)	697	8.5	(6.0–11.8)	8.2	(5.7–11.8)
Low income (100%–199%)	670	7.6	(5.4–10.5)	6.5	(4.7–9.0)
Middle income (200%–399%)	628	7.9	(5.8–10.8)	6.8	(4.7–9.6)
High income (≥400%)	608	10.8	(8.4–13.7)	9.2	(6.7–12.4)
**Body mass index (kg/m** **^2^** **)**					
Normal (<25.0)	583	4.8	(3.0–7.5)	4.3	(2.3–7.9)
Overweight (25.0–29.9)	932	9.4	(7.4–12.1)	7.9	(5.8–10.7)
Obese (≥30.0)	1,053	10.8	(8.7–13.3)	9.9	(7.8–12.3)
**Family history of diabetes**					
Yes	1,013	11.6	(9.5–14.1)	10.4	(8.3–13.0)
No	1,533	7.2	(5.8–8.8)	6.2	(4.8–7.9)
**Medication for hypertension/hypercholesterolemia**					
Yes	1,032	13.4	(11.3–15.9)	13.9	(9.2–20.5)
No	1,571	6.0	(4.8–7.5)	6.1	(4.8–7.7)
**Health coverage status** §§					
Covered	1,995	9.7	(8.3–11.4)	8.4	(6.8–10.3)
Not covered	605	5.5	(3.4–8.7)	4.7	(3.0–7.3)
**No. of doctor visits in the past year**					
<2	888	5.1	(3.7–6.9)	5.4	(3.8–7.6)
2–3	714	9.5	(7.2–12.5)	9.0	(6.5–12.3)
≥4	1,001	11.7	(9.8–14.0)	10.5	(7.9–13.7)
**Usual source for care** ¶¶					
Clinic or doctor’s office	2,062	9.9	(8.5–11.6)	8.9	(7.2–11.0)
Other or none	541	4.2	(2.5–6.9)	4.4	(2.6–7.3)

**Abbreviations**: CI = confidence interval; NA = not applicable.

* Prediabetes awareness defined as adult respondents with prediabetes who 1) answered “yes” to the question, “Have you ever been told by a doctor that you have prediabetes, borderline diabetes, impaired fasting glucose, impaired glucose tolerance, or that your blood sugar is higher than normal but not high enough to be called diabetes or sugar diabetes?,” or 2) volunteered having prediabetes when asked whether they had diabetes.

† Prediabetes defined through laboratory testing (fasting plasma glucose 100–125 mg/dL or hemoglobin A1c 5.7%–6.4%).

§ Total sample size for each category is not the same because of item nonresponse.

¶ Chi-square test of association. All variables, except sex and poverty-income ratio, were significantly associated with prediabetes awareness at p-value <0.05.

** Standardized to the age distribution of the 2000 U.S. Census population. A t-test was used to test differences between subgroups.

†† Includes other Hispanic, multiracial, and others.

§§ Covered included those who answered “yes” to the following questions: “Are you covered by health insurance or some other kind of health-care plan?” and “Do you have Medicare?” (for those aged ≥65 years only).

¶¶ Includes participants who reported having a place they usually go to for care and that this place is a doctor’s office or clinic and those who reported not having a usual source of care or whose usual source of care was hospital outpatient or emergency department.

## Footnotes

* PIR is the household income as a percentage of the poverty threshold income after accounting for inflation and family size and is classified as poor (<100%), low income (100%–199%), middle income (200%–399%), and high income (≥400%).

† Available at http://www.cdc.gov/diabetes/prevention/prediabetes.htm.

§ Additional information available at http://www.cdc.gov/diabetes/prevention.

¶ Additional information available at http://www.yourdiabetesinfo.org.
